# The Clinical Response to Gluten Challenge: A Review of the Literature

**DOI:** 10.3390/nu5114614

**Published:** 2013-11-19

**Authors:** Maaike J. Bruins

**Affiliations:** DSM Biotechnology Center, Alexander Fleminglaan 1, Delft 2613AX, The Netherlands; E-Mail: maaike.bruins@dsm.com; Tel.: +31-613-207-601; Fax: +31-152-794-110

**Keywords:** gluten challenge, coeliac disease, diagnosis

## Abstract

The aim of this review was to identify, evaluate and summarize all relevant studies reporting on the clinical response to gluten challenge by adult or pediatric patients with suspected or diagnosed coeliac disease (CD) on a gluten-free diet. We evaluated the effect of gluten challenge on changes in symptoms, intestinal mucosa histology, and serum antibodies. A systematic electronic search was performed for studies published as of 1966 using PubMed and Scopus databases. In the reviewed studies, doses ranged from 0.2 to 30 g/day of wheat gluten or comprised a gluten-containing diet. The onset of symptoms upon gluten intake varied largely from days to months and did not parallel serum antibody or histological changes. Within 3 months of gluten challenge, 70%–100% of pediatric CD patients became positive for AGA-IgA and EMA-IgA antibodies and 50%–70% for AGA-IgG. A limited number of trials suggest that no more than half of adult patients developed positive AGA-IgA, EMA-IgA, tTG-IgA or DGP-IgA/IgG titers. Approximately 50%–100% of pediatric and adult patients experienced mucosal relapse of gluten provocation within 3 months, which was preceded by increased mucosal intra-epithelial lymphocytes within several days of challenge. A 3-month high-dose gluten challenge should be suitable to diagnose the majority of CD patients. In some cases prolonged challenge may be needed to verify diagnosis. Combination testing for antibodies and mucosal histology may fasten the diagnosis.

## 1. Introduction

Significant health complications may occur when coeliac patients remain on a normal gluten-containing diet. Diagnosis of coeliac disease (CD) should accurately be established before starting a person on a livelong gluten-free diet. In children and adults, diagnostic testing includes blood HLA-DQ2 and HLA-DQ8 testing, histological examination of small-intestinal biopsies and serum CD-specific antibodies [1,2,3,4,5,6,7]. The diagnosis is confirmed by decline in antibody levels after the exclusion of gluten from the diet. Clinical improvement and histological remission are also supportive clinical endpoint to confirm the disease. Gluten challenge is not necessary, except under unusual circumstances [4] where doubt exists about the initial diagnosis; for example, when the patient is on a gluten-free diet or presents with antibodies or complaints but, nonetheless, normal histology. Moreover, failure to respond to a gluten-free diet may raise doubt regarding the initial diagnosis. Examination of mucosal biopsy, however, involves a potential risk of misdiagnosis since it is subject to large method variability [8,9] and moderate-to-poor inter- and intra-observer reproducibility has been shown [10,11,12]. Unfortunately, histological findings in CD are characteristic but not specific as several disorders can produce comparable histopathological changes [13]. Over recent years, more reliable, specific and sensitive serological diagnostic tests and markers have become available. Small bowel histology remains the gold standard for diagnosis. Symptomatic relapse is not sufficient for a diagnosis of coeliac disease in isolation. Particularly in children in whom the initial biopsy was performed before two years of age, a gluten challenge may be necessary because of the risk of misdiagnosis due to confusion with other causes of enteropathy at this age [3]. In patients suspected of CD and following a gluten-free diet, diagnosis may be confirmed by reintroduction of gluten into the diet or by an oral gluten challenge followed by clinical relapse [3,4,7,14].

Currently, the monitoring of parameters during a gluten challenge is largely empirical, particularly in those patients who remain asymptomatic, and the optimum duration and dose of a gluten challenge has not been established yet. Some guidelines propose a gluten diet/challenge until relapse, even for up to 2 years or longer if patients remain symptom free. The ESPGHAN guidelines recommend that daily gluten intake during gluten challenge should contain at least the normal amount of gluten intake for children (approximately 15 g/day) [4,15]. There is considerable inter-individual variability of clinical presentation among patients with CD [16,17] but also in clinical response time to gluten intake [17]. The large variability and lack of predictability in the response time and severity to gluten complicate defining recommendations regarding the duration and dose of necessary gluten challenge in the diagnostic setting as well as the clinical trial setting.

A standardized approach regarding the amount and duration of dietary gluten necessary to provoke a clinical response in children and adults could provide guidance to physicians and investigators. Therefore, the aim of this article was to perform a review of the literature reporting on the course of the clinical symptoms, serum CD autoantibodies, and intestinal histological changes in response to a gluten challenge in children and adults with diagnosed or suspected CD.

## 2. Method

The data sources used for this systematic review of references published between 1966 and July 2013 included PubMed and Scopus. Only publications in English were included. We included studies that evaluated the effect of oral gluten challenge in individuals with CD on clinical parameters, *i.e.*, CD-specific antibodies, histology of small bowel mucosa biopsies, symptoms, and urinary sugar absorption test. We reviewed the studies that described the effect of a gluten challenge on clinical relapse in order to confirm diagnosis of CD in children or adults with diagnosed and/or suspected CD. We also extracted valuable information of patients receiving a gluten challenge in the placebo arm of clinical trials testing CD-related therapies. Gluten challenge studies with the aim to determine the safe threshold of prolonged exposure to trace amounts of gluten were beyond the scope of this paper. Studies reporting on positive anti-gliadin antibodies (AGA), anti-endomysial antibodies (EMA), anti-tissue transglutaminase antibodies (tTG), and anti-deaminated gliadin peptide antibodies (DGP) were included. Anti-reticulin antibodies were not reviewed as this test has nowadays been replaced by the more reliable AGA test. While AGA antibodies have been in use for several decades. However, there is a wide variability in their diagnostic accuracy and both AGA-IgA and AGA-IgG have sensitivities and specificities inferior to tTG-IgA and DGP-IgA and are no longer included in the routine testing strategy for CD [5]. Positivity for CD-specific antibodies was defined as concentrations above the assay cutoff value, which varied among assays used in the different studies. The definition of abnormal mucosa histology of small bowel biopsies also varied depending on the biopsy rating scores used (e.g., villous height to crypt depth ratio, Marsh scores). Clinical symptoms in most studies comprised CD-specific symptoms including vomiting, abdominal pain or distension, obstipation, diarrhea, fatty or loose stool, anorexia, weight loss, and growth failure. Only in the summary table we specified whether symptoms constituted mild, moderate or severe symptoms. Studies that were excluded were studies investigating a single-dose gluten challenge, studies investigating oat challenge, rectal gluten challenge, transamidated, hydrolyzed or digested gluten, gluten-specific peptides, and *ex-vivo* studies. Since gluten intake at baseline is likely to influence the response to a gluten challenge, we excluded studies in which patients were on a normal gluten-containing diet or had positive baseline autoantibodies at start. Trials were categorized into trials enrolling pediatric or adult patients with mean age below 18 years or 18 years and older, respectively. Moreover, less patients suspected of having CD can be expected to respond to gluten than patients with a confirmed diagnosis of CD. Therefore, we classified trials according to “confirmed diagnosis based on a biopsy in the past”, or “diagnosed based on inadequate grounds” referring to as “suspected CD”. If possibly, results were reported separately for subgroups of patients with diagnosed and suspected CD in one study. When the amount of dietary gluten in bread was not reported [18,19,20], we estimated the gluten content, assuming that a slice of bread weighs 25–30 g and contains 8–11 g/100 g of protein [15,21], which corresponds to approximately 2–3 g of gluten [7,22]. In some studies the gluten dose was expressed per kg of body weight. If body weight was not given, estimates were based on WHO child growth charts [23].

## 3. Results

Table 1 gives an overview of the included studies. In total, the following studies were identified that investigated the clinical effect of dietary gluten challenge; 16 trials with pediatric patients with biopsy-diagnosed CD, 13 trials with pediatric patients with suspected CD, 11 trials with biopsy-diagnosed adult CD patients and 3 with adolescent or adult patients suspected of having CD. Of the eleven trials with diagnosed adult patients, five reported on the clinical response to a placebo as part of a clinical intervention study [24,25,26,27,28,29]. In the studies included, a gluten challenge consisted of a gluten-containing diet, wheat-derived food products, wheat flour, or wheat gluten powder. The gluten doses ranged from 0.2 to 30 g/day and duration from 1 day to 8 years.

### 3.1. CD-Specific Symptoms in Pediatric Patients with Diagnosed or Suspected CD

The response rates and onset of symptoms throughout the course of gluten challenge in the different studies was highly variable (Table 1); when a gluten-containing diet was given to children with diagnosed CD, 4% of them developed symptoms within 1–2 of weeks [30]. At least 10 g/day of gluten caused symptoms in 13% of children within 12 h [31], 33% of children within 4 weeks of gluten challenge [32], and 60% of children within 3-months of challenge [33]. When given a gluten-containing diet or 3–15 g/day of gluten, 32% of children experienced symptoms within 4–5 months [34]. Smaller amounts of about 2 g/day of gluten caused symptoms in 4% children on the fourth day and in 25% of children after 6 months [18].

In children with suspected CD, a gluten-containing diet induced symptoms in about 26%–33% of children within between a few days to 13 months of gluten challenge [35,36], a gluten challenge of at least 10 g/day caused symptoms in approximately 24%–42% of children from 4 weeks to several [32,37,38,39] months of challenge, only few patients reported severe symptoms during gluten challenge [38]. A gluten-containing diet providing 5 to 15 g/day of gliadin caused symptoms in 59% of children within 45 days of challenge [40]. About 32% of adolescents with diagnosed or suspected CD who received at least 10 g/day of gluten for 2.4 months to 2 years experienced abdominal symptoms at the time of appearance of antireticulin-IgA [41]. In 70% of the cases, the mucosa relapsed before any symptoms had occurred [41]. Lower doses of 0.2–4.3 g/day of gluten, surprisingly triggered symptoms in 79% and 96% of children within 4 and 15 weeks, respectively [42]. No correlation was observed between time of appearance of symptoms and positive antibodies.

Summarizing, in most studies only few children with diagnosed or suspected CD respond by symptoms to a low or high gluten dose during the first 2 weeks. During prolonged low or high dose gluten challenge 24%–42% of children may experience symptoms, although in three studies higher response rates were reported of 60% [33,40], and even 96% [42]. Large variability exists in time of onset of symptoms during gluten challenge: symptoms appear almost immediately in some children while some do not develop symptoms until several months of challenge or develop no symptoms at all. Symptoms are generally mild to moderate. Some studies indicated that clinical symptoms are a very unreliable indicator of antibody response and mucosal relapse [34,41,42].

**Table 1:** The effect of a gluten challenge in pediatric or adult patients on response rate of clinical symptoms, serology, and histology parameters.

Author (year)	Age Group and Age	Diagnosed/Suspected CD	Time on Gluten-Free Diet	Gluten Type and Dose	Duration of Challenge	CD-Symptoms	CD-Antibodies	Mucosal Immunohistology	Sugar Absorption Test
Mayer *et al*. (1989) [31]	Children 3.5^Mdn^ (1.8–9.6) years	Diagnosed by biopsy (*n* = 37)	≥1 year, 17^Mdn^ month	10 g/day gluten either as biscuit or as powder	60^Mdn^ (14–205) days	Acute symptoms in 13% (4/32) within 12 h and symptoms in 0% (0/31) within ~7 months	Increased AGA-IgA and IgG in 65% (20/31) within 15 days	Worsening histology score by Whitehead in 68% (21/31) at 2 month, 84% at 3 month, and 97% within 2 years	Decreased blood xylose within 15 days, remained low up to 150 day
Packer *et al*. (1978) [33]	Children 9.9^M^ (3.0–15.3) years	Diagnosed by biopsy (*n* =32)	6.5^M^ years (0.25–11.0)	≥10 g/day as 4 slices white bread	Up to 3 month	Symptoms in 60% (19/32) within 3 months		Increase in villous atrophy in 78% (25/32) within 3 months	
Hamilton *et al*. (1972) [18]	Children 7.2^M^ ± 1.5^SD^ years	Diagnosed by biopsy (*n* = 23)	3.8^M^ years	2.25 g/day as wheat gluten followed by1 slice/day of bread or equivalent flour (~2–3 g of gluten ^1^)	6 days 6 days–15 months	Symptoms in 4% (1/23) at 4 day, in 8% (1/12) at 1 month, in 25% (3/12) at 6 months		Mucosal lesions in 7% (1/13) within 6 days, 92% (11/12) within 1 year, and 100% within 15 months	
Mavromichalis *et al*. (1976) [43]	Children 6.5^M^ years	Diagnosed by biopsy (*n* = 23)	6.5^M^ year (1.5–10) (*n* = 11 on gluten-free diet)	20 g/day as gluten-containing diet	4–9 weeks			Worsening histology score (III or IV on scale I–IV) in 100% (11/11) within 4–9 weeks Increased IEL in 100% (11/11) within 4–9 weeks	
Hansson *et al*. (1997) [44]	Children 4^Mdn^ (1–18) years	Diagnosed by biopsy (ESPGAN) (*n* = 57)	(*n* = 20 on gluten-free diet)	gluten-containing diet (dose not mentioned)	2 weeks		Positive -AGA-IgA in 30% (6/20) within 2 weeks and 75% (21/28) within 12 weeks -AGA-IgG 15% (3/20) within 2 weeks and 71% (20/28) within 12 weeks -EMA-IgA 35% (7/20) within 2 weeks and 71% (20/28) within 12 weeks -AGA-IgA cells in peripheral blood in 75% (15/20) within 2 weeks and 86% (24/28) within 12 weeks	Increased IEL density in 25% (5/20) within 12 weeks	
Hansson *et al*. (2002) [20]	Children 4^Mdn^ (1–16) years	Diagnosed by biopsy (ESPGAN) (*n* = 57)	(*n* = 21 on gluten-free diet and *n* = 38 on gluten-containing diet	2–3 slices/day of white bread (~4–9 g/day ^1^ of gluten)	12 weeks		Positive -AGA-IgA in 78% (31/40) within 2 weeks, 89% (41/46) within 12 weeks -EMA-IgA in 45% (18/40) within 2 weeks, 91% (42/46) within 12 weeks -tTG-IgA in 45% (18/40) within 2 weeks, 89% (41/46) within 12 weeks	Increased IEL density in 16% (6/38) within 12 weeks	
Schaad *et al*. (1981) [45]	Children 8.1^M^ (3.1–13.1) years	Diagnosed	1.5–10 years	1 g raw cooked gluten/kg/day (~25 g gluten/day ^1,2^)	30 days			Increased IEL in 100% (22/22) at 30 day	
Scott *et al*. (1980) [19]	Children 5.8^Mdn^ (2.9–8.8) years	Diagnosed by biopsy (*n* = 10)	-	One 20 g-slice/day of bread (~2 g/day ^1^ gluten) followed by gluten-containing diet	1 month of bread followed by gluten-containing diet up to 11 month			Mucosal relapse in 100% (10/10) within 2–11 months (7 months^Mdn^) Increased IEL within 2–11 months	
Bürginn-Wolff *et al*. (1991) [46]	Children 4 months–18 years	Diagnosed by biopsy (*n* = 135)	-	Gluten-containing diet (dose not mentioned)	Up to 15 year		Positive AGA-IgA in 97% (28/29) within 3 months, 85% (73/86) within 1 year, and 49% within ≥3 years EMA-IgA 65% (13/20) within 3 months, 84% (49/58) within 1 year, and 93% within ≥3 years	Abnormal mucosa in 72% (31/43) within 1 month, 94% (31/33) within 7–10 months, 95% (18/19) within 20 months	
Ascher *et al*. (1990) [30]	Children 1.4 ^Mdn^ (0.5–16.5) years	Diagnosed by biopsy (ESPGAN) (*n* = 45)	1 year	Gluten-containing diet (dose not mentioned)	3–31 months	Strong symptoms in 4% (2/45) within 1–2 weeks	Positive AGA-IgA in 90% (38/42) of not-IgA deficient patients within 10 months		
Bodé *et al*. (1983) [47]	Children 2.8^Mdn^ (0.3–15.5) years	Diagnosed by biopsy (ESPGAN) (*n* = 14)	≥1 year	≥10 g/day (type not mentioned)	3 months–2 years		Positive AGA-IgG in 79% (11/14) within 3 months–2 years AGA-IgA in 57% (8/14) within 3 months–2 years		
Danielsson *et al*. (1990) [48]	Children ~2^M^ (1–5.6) years	Diagnosed by biopsy (*n* = 67)	0.9–1.4 years	10 g/day as gluten-containing diet	0.5–4.4 years			Abnormal histology score (II–IV on scale I–IV) in 96% (64/67) within 2 years	
Berg *et al* (1997) [34]	Children ~1 year	Diagnosed by biopsy (*n* = 34)	1–1.5 years	Gluten-containing diet or 3–15 g/day of gluten		Symptoms in 32% (11/34) within 4–5 months		Abnormal histology in 100% (23/23) in patients without symptoms within 4–5 months	
Troncone *et al*. (1994) [37]	Children 7.3^M^ (4.9–9.8) years	Suspected (*n* = 6) and diagnosed by biopsy (*n* = 9)	6 years^Mdn^ (3–8)	10 g/day as biscuits or pasta	30^Mdn^ days (14 days–6 months)	Symptoms in 42% (5/12) within 6 months	Positive AGA-IgA in ~AGA-IgA in ~50% at 2 weeks, ~25% at 1 month, ~50% at 2 month, ~70% at 3 month, 57% within 6 months AGA-IgG in ~10% at 1 month, ~25% at 2 month, ~50% at 3 month, 29% within 6 months EMA-IgA in ~77% (10/13) at 1 month, 93% within 6 months		Increased urinary cellulose/mannitol ratio in 86% (12/14) within 3 months
Korponay-Szabó *et al*. (1997) [38]	Children 5.1^Mdn ^(1.9–15.3) years	Suspected (*n* = 67) and diagnosed by biopsy (ESPGAN) (*n* = 90)	Not reported	5–10 g/day as purified gluten	6 weeks–2 years	-Mild symptoms in 34.3% (46/134) -Severe symptoms in 2.9% (4/134) of patients with histological relapse within 6 weeks–2 years	Positive EMA-IgA or -IgG in 66% at 3 month, 90% at 6 month, and 88% (134/153) within 21 months	Abnormal histology score (scale I-III by Fontaine and Navarro) in 88% (134/153) within 2 years. Relapse time (A + B) 5^Mdn^ month (1.8–26.5) and (C) 6^Mdn^ month (1.4–25.3)	
Rolles *et al*. (1976) [32]	Children 5.7^M^ (1.5–15) years	Suspected (*n* = 35)	4.2^M^ (1–10) years	20 g/day as gluten powder	4–13 weeks	Mild to severe symptoms in 29% (10/35) within 4–13 weeks		Abnormal histology score (scale 3 or 4 on 0–4) in 51% (18/35) within 4–13 weeks	
Lancaster *et al*. (1976) [49]	Children 11.5^M^ (5–16.5) years	Suspected (*n* = 16)	6.9^M^ (1.5–13) years	10 g/day as wheat protein	Up to 24 month			Decrease in Vh in 62% (10/16) within 3 months, in 81% (13/16) within 3–24 months Increased in IEL density in 100% (13/13) within 3 months	
Laurin *et al*. (2002) [42]	Children 3.8^Mdn^ (2.7–8.8) years	Suspected (*n* = 25)	≥1 year	1.4^Mdn^ g/day (0.2–4.3) as gluten-containing diet	13^Mdn^ week (5 week–1 year)	Symptoms in 79% within 4 weeks, 96% (23/24) within 15 weeks	Positive -AGA-IgA in 25% (5/20) within 4 weeks, 75% within 8 weeks -AGA-IgG in 0% (0/19) within 4 weeks, 5% (5/20) within 8 weeks -EMA-IgA in 65% (13/20) within 4 weeks, 75% within 8 weeks -EMA-IgG in 16% (3/19) within 4 weeks, 25% (5/20) within 8 weeks	-Abnormal histology score (3 or 4 on scale 0–4 by Marsh) in 91% (21/23) within 1 year -Increased IEL count in 96% (22/23) within 1 year	
Laurin *et al*. (2003) [50]	Children 3.8^Mdn^ (2.7–8.8) years	Suspected (*n* = 25)	≥1 year	1.4^Mdn^ g/day (0.2–4.3) as gluten-containing diet	Up to 3 month		Positive -AGA-IgA in 90% (16/18) within 8 weeks -EMA-IgA in 90% within 8 weeks		
Valletta *et al*. (1990) [40]	Children 3.8^M^ (2.7–8.8) years	Suspected (*n* = 17)	0.4–8 years	Gluten-containing diet with 5, 10, 15 g/day gliadin at age 1–3, 3–5, and 5–10 years, respectively	20–45 days	-Symptoms in 59% (10/17) -Food refusal in 100% (17/17) within 45 days	Positive -AGA-IgA in 94% (16/17) within 15–35 days -EMA-IgA in 90% within 2 months	-Worsening histology in 94% (16/17) within 25–45 days -Increased IEL score in 100% (17/17) within 25–45 days	
Jansson *et al*. (2001) [51]	Children 2.7^M^ ± 1^SD^ years	Suspected (*n* = 54)	≥1 year	Gluten powder 0–4 weeks: 0.2 g/kg/day (~2.6 g/day ^1,3^) (A) 0.5 g/kg/day (~6.5 g/day ^1,3^) (B) 4–8 weeks: 0.5 g/kg/day (~6.5 g/day) (A and B)	4–8 weeks		A and B: Positive -AGA-IgA in 76% (38/50) at 2 week, 88% at 4 week, 94% at 8 week no difference between A and B -EMA-IgA in 59% (32/54) at 2 week, 65% at 4 week, 67% at 8 week no difference between A and B	A and B: Worsening histology score (≥3 increase on scale 4–16) in 94% (51/54) at 4 week and 100% at 8 week	
Wauters *et al*. (1991) [39]	Children 5.6^M^ (2–16) years	Suspected (*n* = 17)	46^M^ months (10–168)	Gluten powder: 750 mg/kg bw/day (~14 g/day ^4^) with max 20 g/day	3 months	Symptoms in 24% (4/17) within 3 months	Positive -AGA-IgA in 90% (9/10) within 6 weeks, and 100% (7/7) within 12 weeks -AGA-IgG in 90% (9/10) within 6 weeks, and 100% (7/7) within 12 weeks	Villous atrophy in 59% (10/17) within 12 weeks	
Savilahti *et al*. (1983) [35]	Children 1.6^M^ years	Suspected (*n* = 19)	0.7–2.3 years	Gluten-containing diet (dose not mentioned)	0.1–1.1 year	Symptoms in 26% (5/19) within 0.1–1.1 year	Positive AGA-IgA in 73% (11/15) within 0.1–1.1 year	Abnormal mucosa in 95% (18/19) within 0.1–1.1 year	
Rolles *et al*. (1975) [52]	Children 0.5–5.7 years	Suspected (*n* = 16)	0.1–5 years	20 g/day as gluten powder	Up to 1.5 year	Symptoms in 33% (5/15) within 28 days		Histology score (3 or 4 on scale 0–4) in 80% (4/5) within 1.5 years	Decreased blood xylose in 40% (6/15) within 1 day, 67% (10/15) within 2–7 days, and 100% (15/15) within 14–28 days
Bonamico *et al*. (2005) [36]	Children and adolescents 9.2^M^ (5.4–19) years	Suspected (*n* = 24)		Three gluten-containing meals/day (*n* = 24)	up to 2 month	Symptoms after *in vivo* challenge in 33% (8/24) within few days–2 months	Positive EMA-IgA in 63% (15/24) within 2 months	Abnormal histology score (3 on scale 0–3 by Marsh) in 87% (13/15) within 2 months	
Mäki *et al*. (1989) [41]	Adolescents 16.6^M^ (14.3–22.1) years	Suspected (*n* = 9) and diagnosed by biopsy (ESPGAN) (*n* = 20)	~8^M^ years (3.0–16.0)	≥10 g/day as gluten-containing diet	Up to 2 year	Symptoms in 32% (7/22) anti-reticulin positives within 2.4–24 months	Positive -AGA-IgA in 79% (23/29) within 2.4–24 months -AGA-IgG in 62% (18/29) within 2.4–24 months	Lower Vh in 85% (23/27) within 2.4–24 months. 15% (4/27) did not relapse in 2 years	
Lancaster-Smith *et al*. (1975) [53]	Adults	Diagnosed by biopsy (*n* = 11)	4.3 (1–15) years	25 g as single gluten dose (*n* = 8) or 10 or 20 g/day as gluten-containing diet (*n* = 4)	Single-dose (A) 7 days (B)			A: Increased IEL in 24–48 h B: Increased IEL in 100% within 1 week	
Lähdeaho *et al*. (2011) [54]	Adults: 49^Mdn^ (21–68) years	Diagnosed by biopsy (*n* = 21)	11^Mdn^ (2–34) years	1–3 g/day (biscuits) (A) 3–5 g/day (biscuits) (B)	12 weeks	A: Symptoms in 64% (7/11) within 3 months B: Symptoms in 80% (8/10) within 3 months	A: Positive tTG-IgA in 36% (4/11) within 12 weeks B: Positive tTG-IgA in 50% (5/10) within 12 weeks	A: decreased Vh/Cd in 64% (7/11) within 12 weeks A: Increased IEL in 55% (6/11) patients within 12 weeks B: decreased Vh/Cd ratio in 70% (7/10) within 12 weeks B: Increased IEL in 80% (8/10) patients within 12 weeks	
Leffler *et al*. (2012) [24]	Adults 43^M^ ± 14^SD^ years	Diagnosed by biopsy (*n* = 20)	5 years	3 or 7.6 g/day (bread)	2 weeks	Increased symptom severity at 3 day, 1 and 2 week	Positive -tTG-IgA in 25% (5/20) within 2 weeks (increase to 50% 2 weeks post-challenge) -DGP-IgA/IgG in 30% (6/20) within 2 weeks	Abnormal histology score (3 or 4 on scale 0–4 by Marsh) in 68% (13/19) within 2 weeks Increased IEL within 2 weeks	Increase in urinary lactulose:mannitol ratio within 2 weeks
Montgomory (1988) [55]	Adults 40^M^ (17–74) years	Diagnosed by biopsy (*n* = 13 on GFD)	13^Mdm^ (6–27) months	2.5–5 g/day	14 months		Positive AGA-IgA in 17% (11/13) within 3–14 months	Increased Vh within 14 months: no effect Increased IEL within 14 months	
Brottveit (2011) [56]	Adults41^M^ (16–65) years	Diagnosed by biopsy (*n* = 13 on GFD)	13.9 (0.8–31.6) years	40 g/day (four slices bread)	3 days			Abnormal histology score (3 or 4 on scale 0–4 by Marsh) in 23% (4/13) within 3 days	
Daveson (2011) [57]	Adults 44^M^ (25–58) years	Diagnosed by biopsy (*n* = 10 on GFD in control group)	≥6 months	16 g/day (two slices bread)	5 days			Abnormal histology score (3 or 4 on scale 0–4 by Marsh) in 70% (7/10) within 1 week Increased IEL at 1 week	
Cornell *et al*. (2005) [25]	Adults 18–70 year	Diagnosed by biopsy (*n* = 21 on placebo)	Not reported	3 Cracker biscuits (~1.3 g/day gluten)	2 weeks	>5 Episodes of moderate-to-severe symptoms in 33% (7/21) on placebo within 2 weeks challenge and the following 10 week	Positive tTGA >5 U/mL in 19% (4/21) within 2 weeks and 3–15 weeks post-challenge	-Increased lymphocyte score in 83% (5/6) at 2 week -Increased epithelial stunting in 50% (3/6) at 2 week	
Tye-Din *et al*. (2010) [26]	Adults 41^Mdn^ (21–67) years	Diagnosed by biopsy (*n* = 10 on placebo)	≥8 weeks	16 g/day Wheat flour slurry	3 days	Symptoms increased within 1 week, 75% of symptoms were mild	No positive tTGA and DGP-IgA/IgG at 6 day		
Kelly *et al*. (2013) [27]	Adults 18–65 years	Diagnosed by biopsy (*n* = 44 on placebo)	≥6 weeks	2.7 g/day Gluten powder (3 × daily 0.9 g)	6 weeks	Symptoms increased in 80% within 6 weeks. Plateau at 3 week	Positive tTG-IgA > 10 U/mL in 30% (13/44) at 6 week		Increase in urinary lactulose:mannitol ratio. Plateau at 4 week
Leffler *et al*. (2012b) [28]	Adults 18–72 years	Diagnosed by biopsy (*n* = 14 on placebo)	≥6 weeks	2.4 g/day Gluten powder (3 × daily 0.8 g)	2 weeks	Symptoms increased in 50% within 2 weeks	No positive tTG-IgA at 2 week		Increase in urinary lactulose:mannitol ratio at 2 week
Tack *et al.* 2010 [58]	Adults 55^Mdn^ (20–68) years	Diagnosed by biopsy (*n* = 7 on placebo)	7.5 (2–40) years	7 g/day (5 toasts)	2 weeks	Symptoms increased in 43% (3/7) within 2 weeks	Positive -tTG-IgA in 14% (1/7) -AGA-IgA in 14% (1/7) -AGA-IgG in 14% (1/7) No positive EMA-IgA within 2 weeks	-Increased tTG-IgA deposits in 71% (5/7) -Abnormal histology score (3 or 4 on scale 0–4 by Marsh) in 23% (2/7) Within 2 weeks	
Kumar *et al*. (1979) [59]	Adolescents 16.1^M^ (14–21) years Adults: 37.7^M^ (17–59) years	Suspected (*n* = 28)	Adolescents: 6.4^M^ (1–14) years Adults: 2.8^M^ (0.75–7) years	≥4 Slices bread (~10 g/day gluten)	Adolescents 4–17.5 weeks (23^Mdn^) Adults: median 4–25.5 weeks (11.5^Mdn^)	Adolescents: Symptoms in 67% (6/9) within 1 h–2 weeks, no symptoms in 33% within 1 year Adults: Symptoms in 84% (16/19) within 4 days–3 months, no symptoms in 16% within 1 year		Adolescents: Decreased Vh in 56% (5/9) within 8 weeks, 100% within 10 momths Increased IEL in 100% within 10 months Adults: Decreased Vh in 95% (18/19) within 7 weeks, still 95% within 1 year Increased IEL in 95% within 7 weeks	
Wahab (2001) [60]	Adults 40^M^ (16–74) years	Suspected (*n* = 37)		30 g/day on top of GCD	2 months	Symptoms in 55% (17/38) within 3 months	Positive AGA-IgA in 22% (8/37) within 2 months Positive EMA-IgA in 17% (4/23)within 2 months	Abnormal histology score (2, 3 or 4 on scale 0–4 by Marsh) in 32% (12/38) at 2 month	
Kaukinen *et al.* (2005) [61]	Adults 45^M^ (19–70) years	Suspected (*n* = 21)	Not reported	≥15 g/day (5 Slices of bread)	6 months			-Increased tTG-IgA deposits in 24% (5/21)	

Abbreviations: CD: Coeliac Disease, Vh: villous height, Cd: crypt depth, M: mean; Mdn: median; SD: standard deviation. ^1^ Assumption made as described in the Methods. ^2^ Assuming an 8-years old child weighs 25 kg. ^3^ Assuming a 2.7-years old child weighs 13 kg. ^4^ Assuming a 5.6-year old child weighs 19 kg.

### 3.2. CD-Specific Symptoms in Adults with Diagnosed or Suspected CD

In three studies, effects were reported of gluten challenge on symptoms in diagnosed or suspected adult or adolescent CD patients [54,59,62]. In five clinical trials [25,26,27,28,58], the effects of gluten challenge given to diagnosed adult patients in the placebo arm were reported. Reintroduction of a gluten-containing diet induced gastrointestinal symptoms in 77% of patients suspected of CD between 1 and 8 months of challenge and CD was confirmed in 40% of these patients [62]. The diagnosis CD was nevertheless confirmed in 65% of the 33% patients who did not develop symptoms. Symptoms occurred in 67% of patients with confirmed diagnosis of CD. When diagnosed or suspected CD patients received 7 to 10 g/day of gluten, 43% [58] and 67% [59] reported symptoms within two weeks of challenge. Within 3 months 84% had experienced symptoms [59]. Most symptoms occurred after one week of challenge [59]. After a 2-week and 6-week challenge period with about 2.5 g/day of gluten three times daily, about 50% of patients [28] and 80% of patients [27] reported complaints, respectively. The severity of symptoms increased after 2 weeks [27,28] reaching a plateau at 3 weeks [27]. When diagnosed CD patients received a low (1–3 g/day) or a high (3–5 g/day) dose of gluten, 64% and 80% of them, respectively, reported symptoms within 3 months [54]. A 2-week challenge of ~1.3 g/day of gluten triggered symptoms in 66% of patients the following 12 weeks; about 33% had more than five episodes of moderate to severe symptoms [25].

In summary, the number of adult patients reporting symptoms as well as the severity of symptoms may increase throughout gluten challenge. Within 3 months of gluten challenge, about 64%–80% of adult patients can be expected to experience symptoms. A proportion of patients with CD may never develop symptoms during gluten challenge. The onset of symptoms is rather unpredictable. The appearance of symptoms during gluten challenge is no indicator of CD.

### 3.3. Antibodies in Pediatric Patients with Diagnosed or Suspected CD

#### 3.3.1. AGA-IgA and AGA-IgG Antibodies

**Figure 1:**
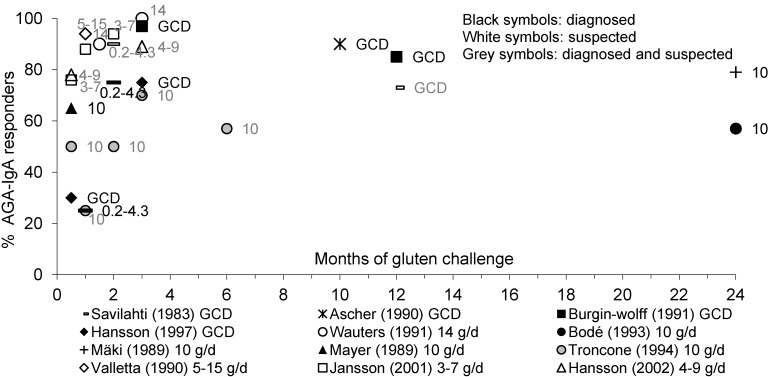
Percentage of pediatric patients with diagnosed or suspected coeliac disease (CD) showing an anti-gliadin antibodies (AGA)-IgA response to gluten over time.

Figure 1 illustrates the time course of children with diagnosed or suspected CD responding to a gluten challenge by positive AGA-IgA antibodies.

The proportion of children with diagnosed or suspected CD responding to gluten challenge by AGA-IgA antibodies varied widely. After 2 weeks, about 30% to 78% of children had responded to a challenge providing 3 to 15 g/day of gluten [20,31,44,63,64]. After 2 to 3 months of challenge with 4 to 14 g/day of gluten, about 70%–100% of children showed positive AGA-IgA antibodies in their serum [20,44,46,63,64]. No clear dose-response effect was observed between the different studies. In two studies with a low dose of gluten (0.2–4.3 g/day), the percentage of children responding by AGA-IgA was 90% [50] or 75% after 2 months [42]. Within 10 months to 1 year, 73%–90% of children had developed AGA-IgA antibodies [30,35,46]. Interestingly, the percentage of CD children with AGA-IgA was highest (97%) after a gluten consumption period of about 1 to 3 months and decreases thereafter to 85% at 1 year, and 49% after 3 years or more of gluten intake [46].

**Figure 2:**
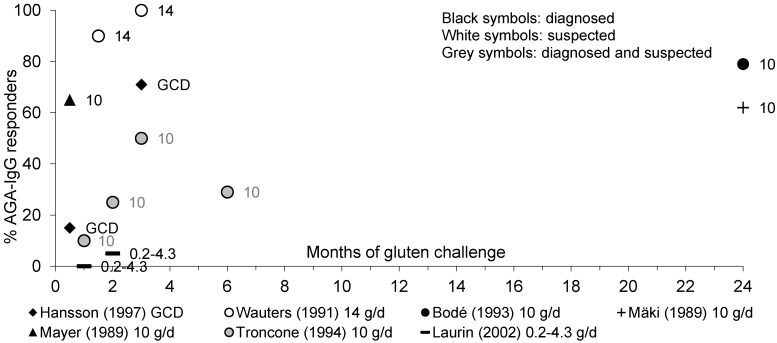
Percentage of pediatric patients with diagnosed or suspected CD showing an AGA-IgG response to gluten over time.

Figure 2 shows the proportion of diagnosed or suspected CD children responding with positive AGA-IgG antibodies to a gluten challenge.

Whereas most studies showed less children responding by AGA-IgG than by AGA-IgA throughout the course of gluten challenge [41,42,44,63], two studies showed similar response rates by AGA-IgA and AGA-IgG [31,39]. When children with CD were given a gluten-containing diet or 10 to 14 g/day of gluten, AGA-IgG rose significantly in 15% [44] or 65% [31] of children within 2 weeks and in 71%–100% of children within 3 months of challenge [39,44]. In two studies in which children received 10 g/day [63] or 0.2–4.3 g/day [42] of gluten, only 25% and 5% of children had responded by AGA-IgA after 2 months of gluten challenge, respectively.

#### 3.3.2. EMA-IgA Antibodies

**Figure 3:**
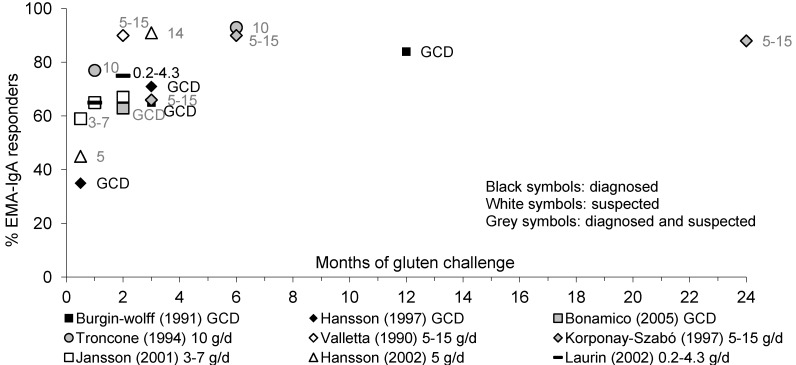
Percentage of pediatric patients with diagnosed or suspected CD showing an anti-endomysial antibodies (EMA)-IgA response to gluten over time.

Figure 3 summarizes the proportion of children with diagnosed or suspected CD developing positive EMA-IgA during gluten challenge.

In a number of trials with children with diagnosed or suspected CD, results on serum EMA-IgA levels were reported during gluten challenge doses from 0.2 to 15 g/day of gluten or a gluten-containing diet. After 2 weeks of challenge, 35% to 59% of children showed positive EMA-IgA antibodies [20,36,40,42,44,46,63,64,65], 65% to 77% of children after 1 month [42,51,63], between 63% and 100% of children became EMA-IgA positive between 2 and 3 months [20,36,38,40,42,44,46,51], while 84% to 93% of children had become positive from 6 months to 3 years of challenge [38,46,63]. Even small gluten amounts caused relapse by EMA-IgA [42]. There was no clear difference in time to EMA-IgA positivity between the different gluten doses.

#### 3.3.3. tTG-IgA Antibodies

In one study, diagnosed CD children received 4–9 g/day of gluten [20]; positive tTG-IgA levels were detected in 45% and 89% of children within 2 and 12 weeks, respectively.

#### 3.3.4. Antibodies in Pediatric Patients: Summary

In summary, the time it takes for children to relapse by antibodies with a gluten challenge is variable. Moderate-to-high gluten challenge doses given to children with diagnosed or suspected CD increased AGA-IgA, AGA-IgG and EMA-IgA to positive levels within the first few weeks. Within 3 months of challenge, the majority of children had developed AGA-IgA, AGA-IgG, EMA-IgA or tTG-IgA antibodies. Only few children relapsed by AGA-IgA, AGA-IgG and EMA-IgA after 1 year. No clear difference in relapse rate to gluten was observed between children with diagnosed and suspected CD. Conversion of AGA-IgA positive to negative tests has been reported to occur in some patients.

### 3.4. Antibodies in Adult Patients with Diagnosed or Suspected CD

#### 3.4.1. AGA-IgA and EMA-IgA Antibodies

In four trials, AGA-IgA antibody titers in gluten-challenged adult patients with suspected or diagnosed CD were reported [26,29,55,60]. The AGA-IgA titers increased in 14% of diagnosed patients in the placebo arm after a 2-week 7 g/day gluten challenge [58] and in 85% of diagnosed patients receiving 2.5–5 g/day of gluten for up to 14 months [55]. Increased AGA-IgA was observed in 22% of borderline patients receiving 30 g/day on top of a normal diet for up to 2 months [60], None of the diagnosed CD patients receiving 16 or 7 g/day of gluten developed positive EMA-IgA antibodies within 2 weeks [29,58]. In borderline patients, 17% became EMA-IgA positive after a 2-month very high-dose gluten challenge [60].

#### 3.4.2. tTG-IgA and DGP-IgA/IgG Antibodies

**Figure 4:**
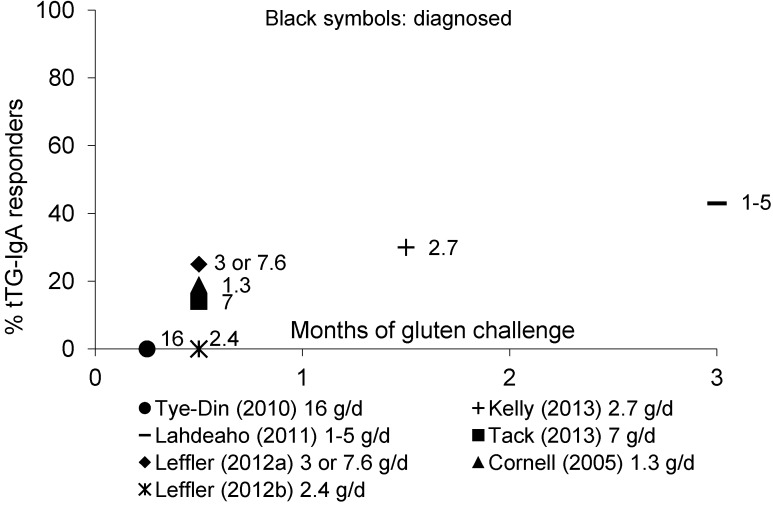
Percentage of adult patients with diagnosed CD showing a tTG-IgA response to gluten over time.

Figure 4 shows diagnosed adult CD patients responding by tTG-IgA throughout gluten challenge.

The effects of gluten challenge on tTG-IgA titers in diagnosed adult CD patients were reported either [24,25,26,27,28,29] or not [54] as part of a clinical trial. No positive tTG-IgA antibodies were observed at day 6 post-challenge in any of the diagnosed CD patients receiving 16 g of gluten for 3 days [26]. After a 2-week challenge with a dose from 1.3 to 7.6 g/day of gluten, tTG-IgA increased in 0% to 25% of adult diagnosed CD patients [24,25,28,29]. Longer gluten challenge form 6 weeks to 3 months increased the proportion of tTG-IgA-positive patients to 30%–43% [27,54]. A 3-day gluten challenge of 16 g/day did not increase DGP-IgA/IgG titers at day 6 [26], but 3 or 7.5 g/day induced positive DGP-IgA/IgG titers in 30% of diagnosed patients within two weeks and 45% the following two weeks [24].

#### 3.4.3. Antibodies in Adult Patients: Summary

In summary, few diagnosed CD patients responded by AGA-IgA, EMA-IgA, tTG-IgA, or DGP-IgA/IgG antibodies after 2 weeks of gluten challenge. Within 6 weeks to 3 months of gluten challenge, still no more than 50% of patients became positive for these antibodies.

### 3.5. Mucosal Immunohistology in Pediatric Patients with Diagnosed or Suspected CD

#### 3.5.1. Mucosal IEL

When children with diagnosed or suspected CD received 5 to 25 g/day of gluten, 91% to 100% of them developed increased mucosal IEL within 1 to 2 months [40,42,43,45,50]. Within 3 months of gluten challenge with 10 g/day, all children with suspected CD showed increased mucosal IEL counts [49]. In one study less children, 16% to 25%, responded with increased IEL within 3 months of about 6 g/day of gluten challenge [20,44]. The authors of one study found that the gluten intake dose strongly correlated with the degree of inflammation in the biopsy, as expressed by IEL [42]. A gluten challenge increased IEL in mucosal biopsies before histological changes occurred.

#### 3.5.2. Mucosal Histology

**Figure 5:**
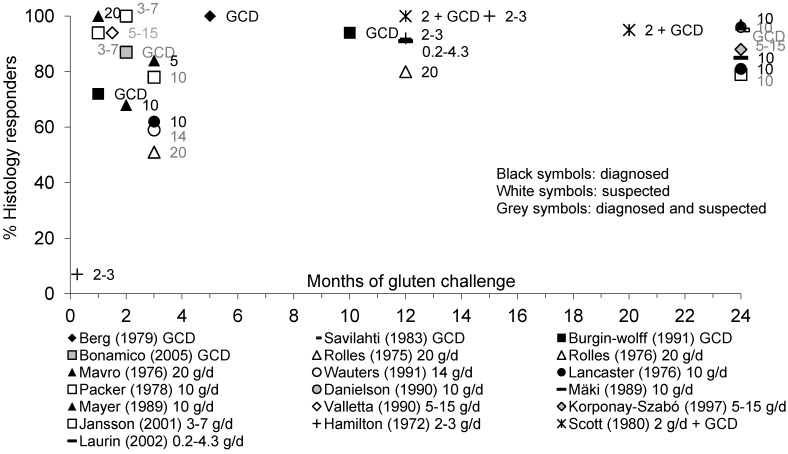
Percentage of pediatric patients with diagnosed or suspected CD showing histological response to gluten over time.

Figure 5 gives an overview of children with diagnosed or suspected CD developing changes in mucosal morphology throughout gluten challenge.

The proportion of children with diagnosed or suspected CD having abnormal mucosal histology gradually increased during the course of gluten challenge. Only 7% of children with diagnosed CD developed mucosal lesions after 1 week when a gluten challenge of 2–3 g/day of gluten was given [18]. However, when doses of 3 to 20 g/day of gluten were given, the proportion of children developing an abnormal small bowel mucosal histology scores within 1 month ranged from 72% to 100% [43,46,64]. Mucosal relapse rates after 2 to 3 months of challenge ranged between 51% and 100% [31,32,33,36,39,49,64]. After 5 months to 2 years of gluten challenge, the majority of children have relapsed by mucosal abnormalities, with relapse rates of 79% to 100% reported in the different studies [18,19,31,33,34,41,42,46,48,49,52]. After 1 year low-dose gluten challenge (0.2 to 4.3 g/day gluten), the proportion of children showing abnormal small bowel mucosal histology scores did not differ from those receiving a higher dose challenge (5 g/day or higher). For some children it took 2 or even 8 years to relapse on gluten [48]. As expected, the intestinal mucosa relapse rate was higher in diagnosed than in suspected patients: within 3 months of gluten intake, respectively, about 60% and 80% had relapsed.

#### 3.5.3. Mucosal Immunohistology: Summary

In summary, within 1 month of gluten exposure, mucosal IEL counts were increased in almost all children with diagnosed or suspected CD. The percentage of children developing moderate to severe mucosal histological abnormalities within 2 to 3 months of gluten challenge ranged between 51%–100%. When child patients are biopsied after one week of challenge, only a minority show morphological relapse. The majority of children will have relapsed after 2 to 3 months of challenge, and only few children relapse thereafter.

### 3.6. Mucosal Immunohistology in Adult Patients with Diagnosed or Suspected CD

#### 3.6.1. Mucosal IEL

A single 25-g gluten challenge given to adult patients with proven CD increased IEL in the mucosal biopsy as soon as 24–48 h following challenge [49]. A one-week 10 to 20 g gluten-containing diet increased IEL density in the mucosal biopsy of all patients [49,57]. Lower gluten doses (3–7.6 g/day) also increased mucosal IEL of patients within 2 weeks [24]. Gluten challenges of 10–25 g/day increased mucosal IEL counts in 95% to 100% of adult or adolescent patients with diagnosed or suspected CD within 1 to 2 months [59], and 3 to 14-months [55,59]. Increased IEL were found in 55% and 80% of diagnosed CD patients receiving, respectively, 1–3 g/day and 3–5 g/day of gluten for 3 months [54].

#### 3.6.2. Mucosal Histology

**Figure 6:**
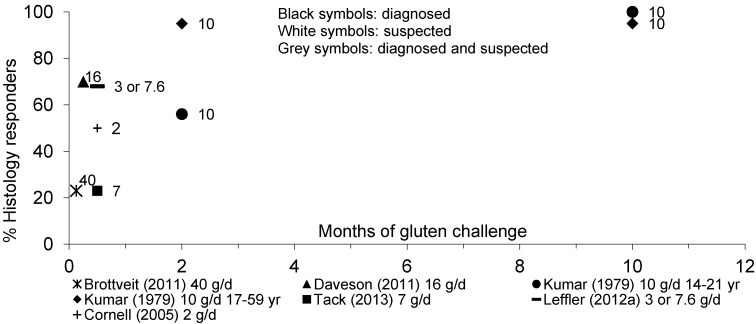
Percentage of adult patients with diagnosed or suspected CD showing histological response to gluten over time.

Figure 6 illustrates the proportion of adult diagnosed or suspected CD patients responding by abnormal small bowel mucosal histology throughout a gluten challenge.

Increased biopsy Marsh scores were observed in 23% of diagnosed CD patients receiving 40 g/day of gluten for 3 days [56], and in 70% of diagnosed CD patients receiving 16 g/day of gluten for 5 days [57]. Gluten doses between 1 and 7 g/day induced abnormal histology scores in 23% to 68% of adult diagnosed CD patients within 2 weeks [24,25,29], and 67% of patients within 3 months [54]. In adolescents and adult patients with suspected CD, 10 g/day of gluten triggered mucosal relapse in 56% and 95% of them within 2 months, and 95% and 100% of them within 1 year, respectively [59]. Less patients with borderline CD may respond to gluten; 32% showed abnormal histology scores within 2 months of high-dose gluten challenge [60].

#### 3.6.3. Mucosal tTGA-IgA Deposits

A gluten challenge of at least 15 g/day for 6 months induced positive tTGA-specific mucosal IgA deposits in 24% of suspected CD patients [61].

#### 3.6.4. Mucosal Immunohistology: Summary

In summary, the results of gluten challenge on mucosal histology in adult patients are variable. More than two weeks of high-dose gluten challenge may be required to induce small intestinal mucosal morphology changes in the majority of patients. However, IEL can appear as early as 1 to 2 days after gluten challenge with increased counts in all patients after 4 weeks. Mucosal tTGA-IgA deposits is another marker appearing in the majority of patients within 2 weeks of challenge.

## 4. Discussion

### 4.1. Strength and Weaknesses

To our knowledge, this is the first review giving an overview of gluten challenge studies in patients suffering from CD or suspected of having CD and the consequences on symptoms, mucosal damage and CD-specific antibodies. In this review, we excluded studies with patients who were on a regular gluten diet at the time of challenge, as their response to gluten may be lower and not representative for patients on a gluten-free diet. This review has, however, several limitations. The gluten challenges used in all studies were wheat-derived and hence, findings relate to wheat gluten. There is very limited data looking at the effect of barley hordein or rye secalin on CD outcomes in the published literature (e.g., [66,67]), but evidence exists that these prolamins induce effects different to wheat gluten, at least at an immunologic level. Moreover, the quoted gluten amounts in the publications were mostly estimates and probably not accurate. In a few studies, gluten amounts were analyzed by R5 ELISA probably providing better estimates. Although several studies looked at the effect of gluten challenge in pediatric patients, the number of studies with adult patients is limited. The clinical response to gluten is most likely larger in diagnosed CD patients than in patients suspected of having CD in whom part may not have CD. Nevertheless, the results for both groups were combined in the figures. Furthermore, the participants in the different studies convey a heterogeneous group with respect to age, gluten dose, and time on a gluten-free diet, and criteria for diagnosis and are therefore difficult to compare. Also methodologies for measurement of antibodies, biopsies, and histology were different including the cutoff levels used to define antibody or histological positivity. Another limitation is that in most studies in the seventies to nineties, AGA-IgA and AGA-IgG antibodies were most commonly measured. However, particularly AGA-IgA has a poor sensitivity compared to newer antibodies such as EMA-IgA, tTG-IgA, and DGP-IgA/IgG which may have resulted in an underestimation of the patients responding to gluten by positive AGA-IgA titers.

### 4.2. Occurrence of Symptoms in Response to Gluten

Until recently, no proper guidelines for categorizing symptoms were available, making it difficult to compare the symptoms reported in the different studies. Moreover, symptoms in response to gluten are not CD-specific as approximately half of non-coeliac patients also show exacerbation of symptoms during gluten challenge [62]. Gastrointestinal symptoms are not specific for CD. The predictive value of symptoms after gluten re-introduction or gluten challenge is very low [34,38,39,41,62]. In one study the positive predictive value of symptoms for having CD was 52% [62]. In diagnosed adult patients, the symptom response rate seems to range somewhere between 65% and 85% [26,59] and most symptoms seem to occur within 1 to 2 weeks [26,59]. Less children (24%–42%) than adults (64%–80%) reported symptoms throughout prolonged gluten challenge but this may strongly depend on the methodology used in the different studies.

In summary, symptoms upon gluten challenge are hard to predict and have low positive predictive value. Recently, a validated disease-specific symptom index for coeliac disease was developed, but it remains to be established whether this can be used as an independent outcome measure for the monitoring of coeliac disease [68].

### 4.3. Occurrence of Antibodies in Response to Gluten

The CD-specific antibody and mucosal response is more predictable than the appearance of symptoms. Nevertheless, considerable variation between patients exists in the time to serological relapse on gluten [17]. On average, about 70%–100% of diagnosed pediatric CD patients given a moderate to high-dose gluten challenge will have responded by AGA-IgA, EMA-IgA, and tTG-IgA antibodies within 3 months of moderate-to-high gluten intake. Less children responded to gluten by AGA-IgG than by AGA-IgA. Compared to diagnosed patients, slightly less patients suspected of CD developed positive antibodies, but the majority had responded by 3 months. Also low dose prolonged gluten challenge caused serological or histological relapse in children with (suspected) CD [25,69]. In these studies, mucosal changes to gluten correlated with the gluten dose given, suggesting a dose-dependent response to gluten. Histological relapse occurred faster in children receiving a larger gluten dose in children with diagnosed CD [51,70], also suggesting a dose-response effect. Therefore, when testing serological antibodies during gluten challenge of approximately 15 g/day on a 3 to 6 monthly basis as recommended by the current ESPGHAN recommendations [4], most cases of CD should be detected. While the majority relapses in three months, for a few patients it may take longer to relapse, and in rare cases it may take years to relapse. Conversion to antibody negativity during prolonged gluten intake has been reported, suggesting that in rare cases gluten tolerance may develop [46,71].

In adult patients, the few available studies suggest that no more than half of the patients develop positive serum antibodies (AGA-IgA, EMA-IgA, tTG-IgA, and DGP-IgA/IgG) in response to a 6-week to 3-month gluten challenge. The few available studies suggest that the AGA-IgA and EMA-IgA response rates of adult CD patients to high-dose gluten challenge was very low. This suggests a lower response in diagnosed adult than pediatric patients. Whether this lower antibody responsiveness to gluten in adults is due to a longer period of gluten withdrawal remains to be established.

### 4.4. Occurrence of Histological Changes in Response to Gluten

About 50% to 100% of children with diagnosed or suspected CD developed moderate to severe mucosal histological abnormalities within 2 to 3 months of gluten challenge. Comparable response rates were reported for adult patients. As can be expected, the average 3-month relapse rate in patients with diagnosed CD was generally higher than those with suspected CD. Some patients may still show histological relapse on gluten challenge continuing up to 1 or 2 years.

The earliest stages of gluten challenge include increased density of IEL in the mucosa, crypt hyperplasia, and finally, the development of villous atrophy [72], which was confirmed by the reviewed data. The gluten challenge studies showed that mucosal IEL infiltrates respond fast to gluten (days to weeks) whereas the CD-associated antibodies and mucosal morphological deterioration appeared later within weeks to years. In some studies, relapse by abnormal histology of the small bowel biopsy paralleled positive antibodies [38,40,41,51]. In other studies, antibodies and primarily AGA-IgA preceded the worsening of mucosal histology [30,31,39,46] whereas in one study the mucosal changes preceded serum EMA-IgA positivity during gluten challenge [36]. The AGA-IgA antibodies may appear earlier than EMA-IgA during gluten challenge [37,46]. Both in adults and children, symptoms were unpredictable and did not coincide with histological or serological relapse. Within one month of gluten challenge, serological and histological relapse does not occur in all cases during challenge [31,32]. In contrast, increased mucosal IEL were reported in almost all diagnosed pediatric and adult patients within 1 month of gluten challenge [43,45,49,53]. High IEL counts in the mucosa are therefore a fast and sensitive marker of responsiveness to gluten although not specific for CD [42,73,74,75]. In addition, mucosal tTG-IgA deposits are considered to appear rapidly in response to gluten and are both a sensitive and specific marker of early stage CD present in biopsy samples with normal mucosal architecture [61,76]. Although not reviewed in this paper, tetramer staining of gluten-specific T-cells may be supportive in the diagnosis of CD due to the fast appearance after start of a gluten challenge [77].

## 5. Conclusions

To diagnose pediatric patients with suspected CD on a gluten-free diet, a moderate-to-high dose gluten challenge for up to 3 months should be sufficient to induce changes in mucosal histology and antibodies in the majority of patients. In adults on a gluten-free diet, histological and serological relapse rates to gluten may be slower and prolonged challenge may be considered if no relapse is observed. Moreover, testing for combinations of conventional and new early markers with high sensitivity and specificity will significantly shorten the time of gluten challenge to diagnose CD.
